# Skin Grafting En Masse

**Published:** 1879-03

**Authors:** 


Skin Grafting en Masse.—At the sixth congress of the German Surgical Association, Dr. Von Zehender presented a case of a boy, five years old, upon whom he had operated for an ectropium, involving both upper eyelids, resulting from ulceration of supra-orbital structures, due to caries of frontal bone. A piece of skin six centimetres long and three centimetres wide ($2\frac{3}{8}$ by $1\frac{1}{8}$ inch) was removed from the upper arm, the subcutaneous areolar and connective tissue carefully scraped off, until it was smooth, thin and flexible like glove leather. This was then laid upon the clear granulating surface of the eyelids, and the eye was closed for four days by five cat-gut sutures passed through the edges of the lids. The transplantation was entirely successful. While usually taught that sections of skin for grafting should not exceed one square centimetre, this piece contained eighteen square centimetres.—*Centralblatt fur Chirurgie.*

